# On how the mechanochemical and co-precipitation synthesis method changes the sensitivity and operating range of the Ba_2_Mg_1-x_Eu_x_WO_6_ optical thermometer

**DOI:** 10.1038/s41598-021-02309-9

**Published:** 2021-11-24

**Authors:** Quan T. H. Vu, Bartosz Bondzior, Dagmara Stefańska, Natalia Miniajluk-Gaweł, Maciej J. Winiarski, Przemysław J. Dereń

**Affiliations:** Institute of Low Temperature and Structure Research, Polish Academy of Sciences, Okólna 2, 50-422 Wrocław, Poland

**Keywords:** Physical chemistry, Thermoelectric devices and materials, Sensors and biosensors, Electronic structure

## Abstract

The suitability of Ba_2_MgWO_6_ (BMW) double perovskite doped with Eu^3+^ for the construction of an optical thermometer was tested. It has been shown that by controlling the conditions of BMW synthesis, the sensitivity of the optical thermometer and the useful range of its work can be changed. Pure BMW and doped with Eu^3+^ samples were prepared using the mechano-chemical and co-precipitation methods. Both the absolute sensitivity and the relative sensitivity in relation to the synthesis route were estimated. The findings proved that the relative sensitivity can be modulated from 1.17%K^−1^ at 248 K, to 1.5%K^−1^ at 120 K for the co-precipitation and the mechanochemical samples, respectively. These spectacular results confirm the applicability of the Ba_2_MgWO_6_: Eu^3+^ for the novel luminescent sensors in high-precision temperature detection devices. The density-functional theory was applied to elucidate the origin of the host emission.

## Introduction

The A_2_MM’O_6_ double perovskite family is one of the most interesting groups of new materials due to their diverse structural and physical properties, as well as promising applications as luminescence^1–3^ and photocatalytic materials, microwave dielectric ceramics^4,5^, and an optical thermometer^2,6^. The chemical formula of a double perovskite is described as A_2_MM’O_6_, where A is an alkaline-earth ion, coordinated by eight to twelve oxygen atoms, M is a divalent metal ion and M’—a hexavalent Mo or W transition metal ion. The M and M’ cations are both coordinated by six oxygen atoms, forming an alternating arrangement of MO_6_ and M’O_6_ octahedra. Double perovskites may form different types of lattices: cubic, tetragonal, orthorhombic or monoclinic—depending on the degree of distortion and deviation from the ideal structure of a cubic perovskite^1–3,7–10^. The attractiveness of perovskites results from the great sensitivity of their structure to external conditions. For this reason, it is interesting to modify their luminescent properties and thermal sensing applicability by synthesis routes. There is no doubt that the optimization of the synthesis methods, as well as the total or partial substitution of host cations in double perovskite materials, by cations with different ionic radius, has a significant impact on their physicochemical properties, including morphology, grain size, and luminescence^11–15^.

The aim of our research was to verify how the morphology of the samples, their shape, size and agglomeration in particles affect the optical properties of this double perovskite, as well as the performance of the luminescent thermometer built applying this host. Eu^3+^-doped materials have been well-known as bifunctional materials for white-light emitting phosphors and field emission displays for years^16–18^. Besides, a few novel hosts doped with Eu^3+^ have been investigated as a temperature readout^2,10^. The investigation was conducted on the example of Ba_2_MgWO_6_ (BMW), undoped and doped with 5% Eu^3+^. To achieve this goal, two different synthesis methods were used, namely mechanochemical (MC)^10^ and coprecipitation (CP)^1^. The synthesis methods adopted, one being related to the solid chemistry route and the other one to the soft chemistry route, lead to obtaining materials of completely different morphologies, although both give samples of pure phase described by the same chemical formula and possessing the same XRD pattern.

The mechanochemical method is a combination of mechanical and chemical processes, it consists of three stages, including mechanical milling, mechanical melting and reaction milling. This causes particle deformation, cracking and welding^19^. The main advantages of this high-energy milling method are, above all, simplicity and availability, lower sintering temperature and higher density, as well as improved microstructure as compared to the commonly used solid-state method^10^.

The co-precipitation method is expected to produce material with smaller crystallite sizes and more homogeneous morphology. In addition, the lower sintering temperature helps to reduce energy consumption^1^.

To the authors’ best knowledge, currently there are only six articles describing spectroscopic properties of the BMW host^1,3,4,10,20,21^. Four of them describe the luminescent properties of BMW doped with lanthanides ions^1,3,4,10^. One focuses on the spectroscopic properties of Ce^3+^ doped ceramics^20^, and one that concerns the luminescence of BMW: Sm^3+^/Dy^3+^ ions^21^. In the 1970s, the emission from the undoped BMW matrix was studied^22,23^. Recently, the enormous potential of using BMW: Eu^3+^ in luminescent thermometry has been presented^10^.

This article is the first one to offer in-depth knowledge on how the morphology of BMW: Eu^3+^ double perovskite, crystallite size, and their agglomeration, influenced by the choice of synthesis method, affect the characteristics of the emission, the energy transfer mechanism between the BMW host and the dopant, and the temperature sensing performance of BMW: Eu^3+^.

It was found that the shape and character of the emission of the Eu^3+^ did not depend on the BMW synthesis method adopted. The samples obtained by both methods exhibited the ^5^D_0_ → ^7^F_1_ magnetic dipole transition associated with vibronic bands.

On the other hand, the emission of the host depends on the method of synthesis. In general, it consists of two bands where the one with higher energy is assigned to regular WO_6_ groups, and the other one to irregular groups^22,23^. The previous work has shown that the emission of regular WO_6_ groups at BMW disappears at room temperature^23^. This paper shows that the BMW sample prepared by the co-precipitation method exhibits the emission of the regular WO_6_ group at 300 K. The origin of such behaviour is explained. Moreover, our density-functional theory (DFT) calculations confirmed the old hypothesis on the origin of two emission bands. Indeed, those which have lower energy originate from the WO_6_ group where W^6+^ enters the Mg^2+^ site.

This article also explains the mechanisms of energy transfer between the regular and irregular groups of WO_6_. It shows that the energy transfer from the BMW matrix to Eu^3+^ ions occurs only from regular WO_6_ groups and proposes a clear model of these processes. It was also demonstrated that the spectroscopic properties of BMW: Eu^3+^ depend on the synthesis method and thus on the sample morphology. The newly discovered features of the BMW host emission can be used to build a luminescent thermometer.

## Results and discussion

BMW double perovskites crystallize in the cubic structure with the *Fm-3 m* space group^1,3,10,20^ in which large Ba^2+^ cation is coordinated by twelve oxygen ions, while Mg^2+^ and W^6+^ are surrounded by six oxygen forming octahedra. The visualization of the crystal structure of the BMW with its detailed description has been introduced in recent publications^3,10^. However, it should be mentioned that in this host Eu^3+^ ions occupy only one crystallographic site with high symmetry (O_h_) of Mg^2+^ ions^3,10^.

The X-ray powder diffractograms (XRD) of the representative samples of BMW: Eu^3+^ were indexed following the pattern ICSD 024–982 (Fig. S1 a, Supplementary information) with lattice constant a = 8.112 Å, cell volume V = 533.81 Å^3^ and Z = 4. A very small amount of impurities was detected as a secondary phase and assigned to Ba_3_WO_6_ (29.4°) for CP and BaWO_4_ (26.4°, 28.1°) for MC samples.

It can be observed that the synthesis technique has a huge impact on the crystallinity. The first difference among various synthesis methods, easily recognizable from the XRD results, is the full-width at half maximum (FWHM) of diffraction peaks which indicate the crystallinity of the samples. Taking into account the peak of the highest intensity at an angle of 2 $\theta$ = 31.16^o^ (Fig. S1 b), it is observed that higher sintering temperature enhanced the crystallinity of the samples. The CP sample with the FWHM = 0.13711 $\pm$ 0.00731 indicates lower crystallinity, due to the lowest sintering temperature (1150 °C for 6 h), as compared to the MC sample (sintered at 1300 °C for 8 h) with a very sharp diffraction line of FWHM of 0.09205 $\pm$ 0.00731. The smaller width of the lines and better crystallinity of MC are the result of the high-energy milling step before annealing.

**Figure 1:**
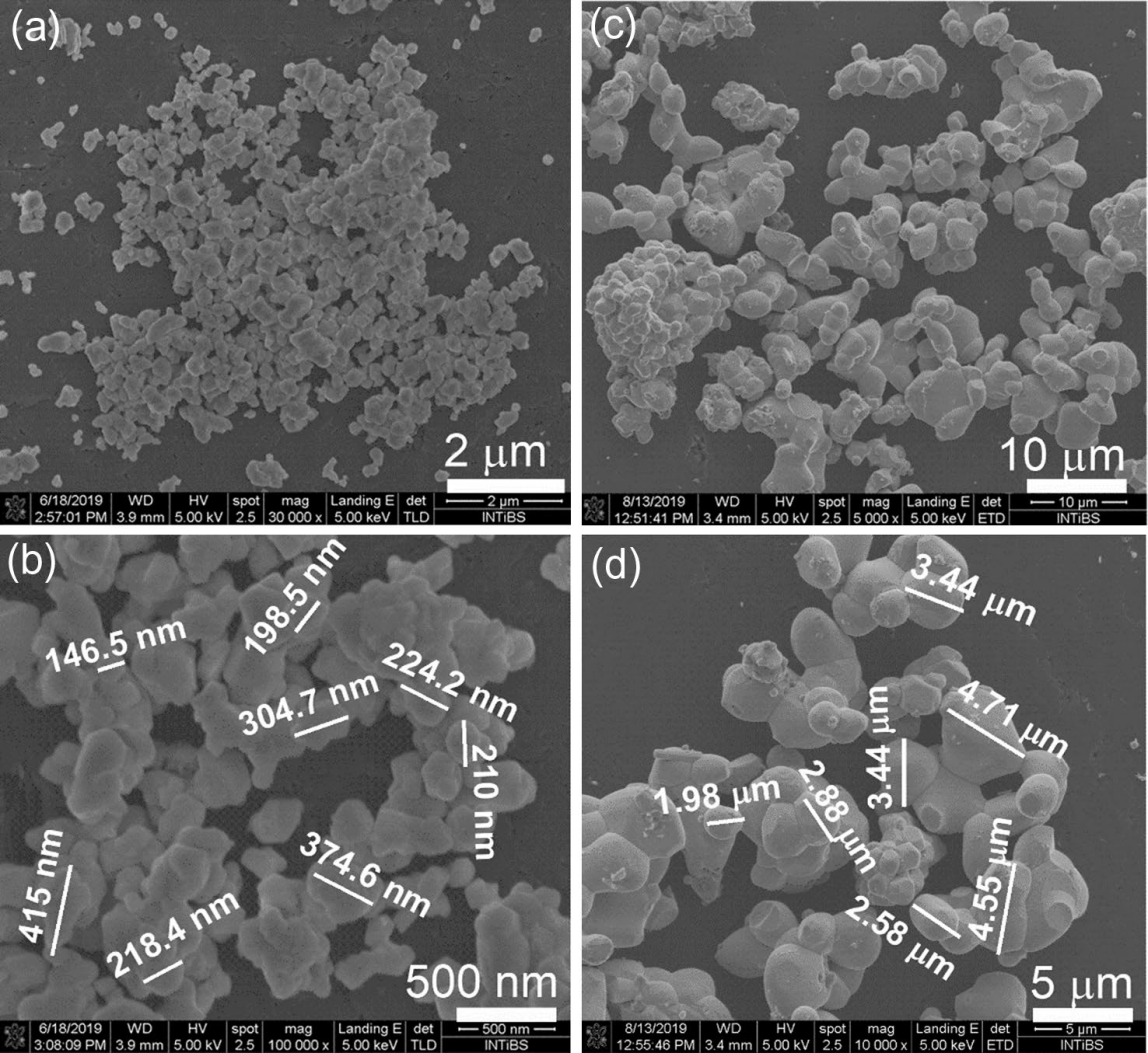
SEM images of BMW: 5% Eu^3+^ synthesized by CP (**a**,**b**) and MC method (**c**,**d**).

The crystallites synthesized with the MC method are larger than the CP ones (see Fig. 1). It is possible to find crystallites in the form of a polyhedron, so their size is slightly smaller than a micrometre. However, most crystallites are larger, ranging in size from 2 to over 6 µm, and have an irregular shape resembling tubers, sometimes with a visible crystal face, indicating the uniform growth of the crystallites in all directions. Most of them are agglomerated to form clusters of two, five, or more particles.

The smallest crystallites were obtained using the co-precipitation method. They are all connected to each other forming clusters, sometimes they are stacked. The average crystallite size is around 200 nm (see Fig. S2)^1^, and the size range is also much smaller than in the case of the MC method, i.e. 20–400 nm. The solid-state method produces irregular polyhedra glued together to form larger particles. The size dispersion of the crystallites is much smaller than for the MC method, but twice as large as for the CP method, and their average size is about 1 µm.

From the highest-magnification micrographs, it seems that the densification occurred only in the MC sample. SEM images resemble those obtained for ceramics in which there are no voids between crystallites. It was evidenced by well-defined boundaries between grains. Besides, some individual grains and porosity are apparently visible. In comparison with the CP sample, the grains are strongly aggregated and the boundaries between the crystallites cannot be clearly identified. By contrast, in the CP samples, there are more individual grains as if they were stacked, which may indicate their flat character. They are seen as if they were growing more intensely only in two directions. More details are shown in the Supplementary Materials (see Fig. S3).

For distribution determination, 100 particles per method were randomly measured from SEM images using the ImageJ software. The MC samples are characterized by a wide distribution in the micrometre scale, whereas the CP samples show a narrow particle size distribution (see Fig. S2). The higher temperature sintering is applied, the larger objects are formed. The size distribution of the MC ones ranged from 1 to 6 µm depending on the Eu^3+^ concentration^10^. The SEM images confirmed the aforementioned conclusion from the XRD results that the MC samples exhibited the highest crystallinity.

For the purpose of comparison, the luminescence of BMW: 5% Eu^3+^ synthesized by distinct methods was measured under 266 nm excitation wavelength at 300 K (Fig. S4a) and 77 K (Fig. S4b). The shape of the Eu^3+^ emission spectra of the MC and CP samples is the same, it does not depend on the synthesis method. The main emission peak is assigned to the magnetic dipole transition between the ^5^D_0_ and ^7^F_1_ levels. The MC sample exhibits a higher emission intensity due to its larger grain size, which was expected since, generally, the higher the crystallinity of the sample, the more intense emission^24^.

**Figure 2:**
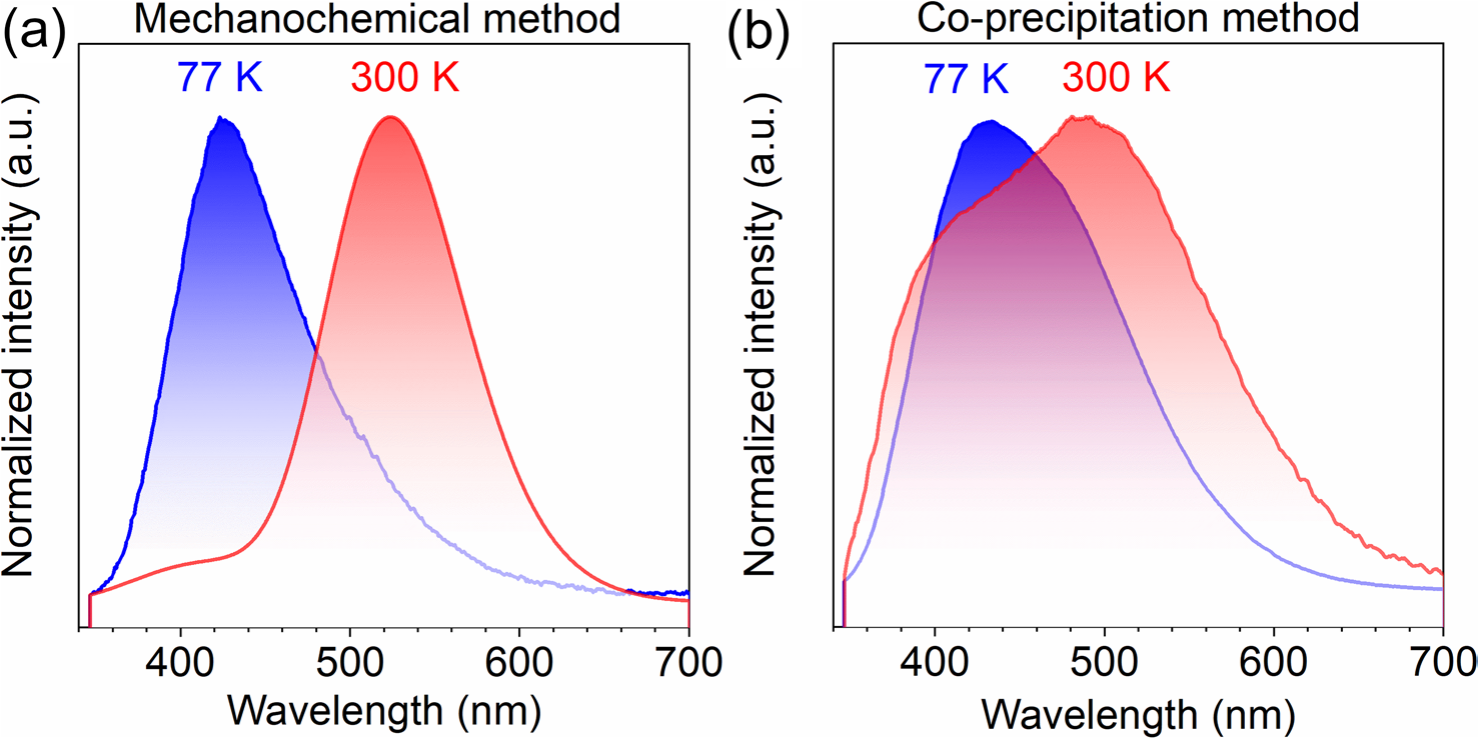
77 K (blue line) and 300 K (red line) normalized emission spectra of undoped BMW samples synthesized by MC (**a**), CP (**b**) recorded under 266 nm excitation.

As was shown in^10^, due to the efficient WO_6_ → Eu^3+^ energy transfer in the BMW: Eu^3+^ samples, the emission is dominated by the latter ions. Therefore, in order to correctly compare the emission spectra of the WO_6_ groups, the measurements of undoped BMW samples were made, they showed wideband emission in the range 350–700 nm. The 77 and 300 K emission spectra of the MC sample cannot be confused. At 77 K, the sample shows a blue-green band with a maximum at 420 nm, while at 300 K there is a yellow-green band with a maximum at 540 nm (see Fig. 2a). The emission of the CP sample combines these two bands into one either at 77 or 300 K, so the emission band is wider than that of the MC sample. The most important is that at 300 K, the blue-green emission band disappears in the MC sample, while in the CP sample is still present (see Fig. 2b).

Of all known tungstates, only the BMW host exhibits two emission bands of the WO_6_ groups and the blue-green one was shown to disappear at 300 K^22,23^, however, the behaviour of the CP sample exhibits a completely new feature—two components either blue-green or yellow-green are observed at once, both at 77 and 300 K. Moreover, this time the blue-green component at 300 K is not only present, but it is more intense than the yellow-green one. At liquid nitrogen temperature, the green component is more intensive than the other part of the spectrum. Only the MC sample behaves as described previously by Blasse and Corsmit^23^.

The blue-green and yellow-green emission bands were assigned to the emission of regular and irregular WO_6_ groups^22^, and it was proposed that tungstate ions may enter not only their regular site in BMW but also may replace Mg^2+^ in the BMW host. This hypothesis was addressed by applying ab initio calculations.

**Figure 3:**
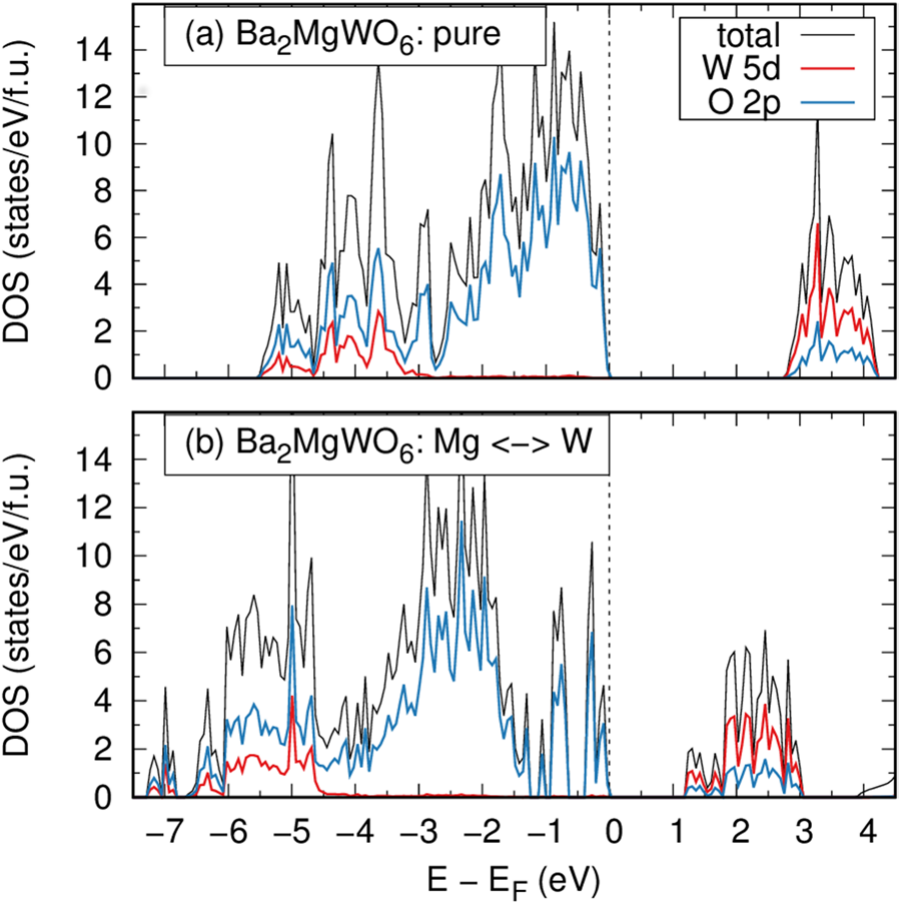
Density of states calculated for (**a**) pure and (**b**) defected (the Mg ↔ W antisite defect) BMW double perovskite oxide.

The density of states (DOS) plots obtained for the parent BMW material are presented in Fig. 3a. The valence band region of this compound is dominated by the O 2p states. The contributions of the W 5d states are present in the valence region of higher binding energy (~ 3 eV below E_F_) and in conduction bands. The p-d bandgap (E_g_) of 2.80 eV, calculated here within the LDA approach, is lower than the experimental one (3.45 eV). It is worth recalling that underestimated E_g_ values were also reported for this system in the recent DFT + U studies^25,26^, whereas the hybrid DFT calculations yielded generally overestimated Eg for double perovskite oxides^27^. However, the calculation of the BMW bandgap was not the main goal of this research. Our aim was to discover whether introducing W to the Mg site would produce an additional band.

The influence of the Mg-W antisite defect on the electronic structure of the material studied in this work is very clear. As depicted in DOS plots in Fig. 3b, in such a case, numerous p- and d-type bands are formed inside a band gap when compared to that of the regular BMW host compound. The presence of additional occupied O 2p bands causes a shift of E_F_ with respect to that of pure BMW. The bandgap of the defected material is also strongly reduced due to the additional W 5d contributions located below the conduction band minimum when compared to that of the host system. The bandgaps of the defected material become narrower so it is also possible to observe their influence on the efficiency of the energy transfer in the BMW host.

**Figure 4:**
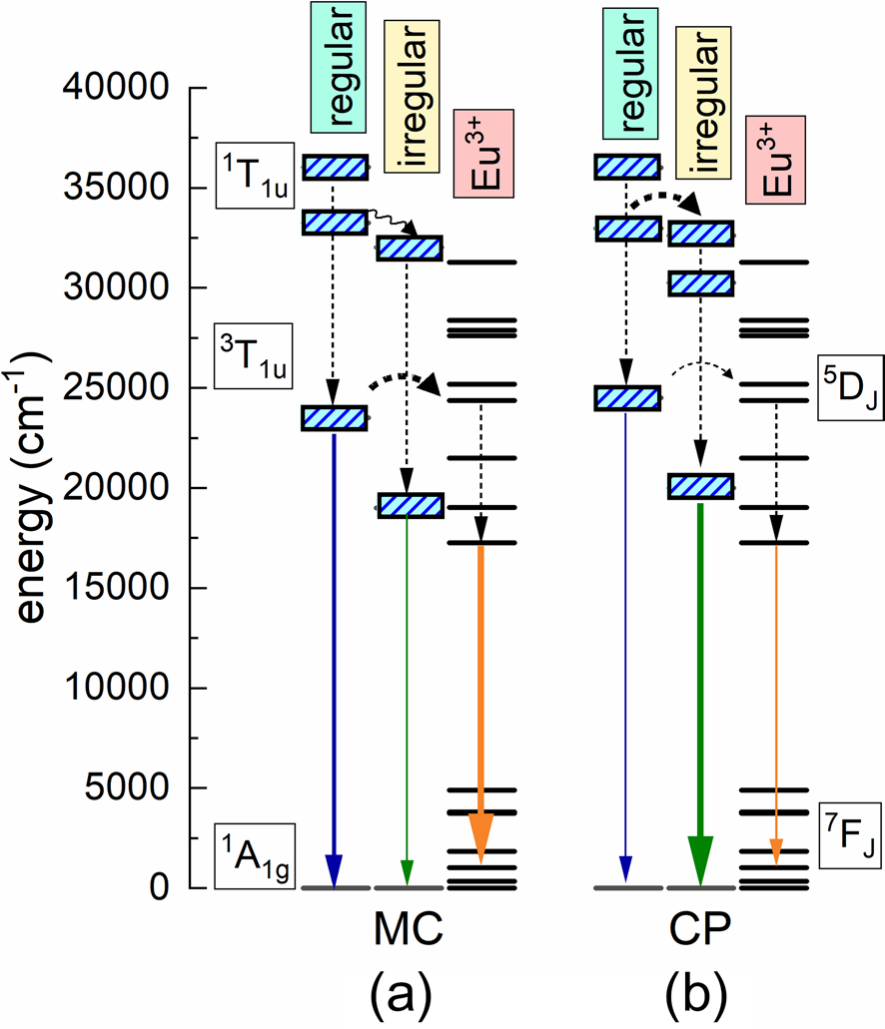
Energy scheme of regular and irregular WO_6_ groups, Eu^3+^ and the energy transfer processes, emissions, and nonradiative transitions occurring in BMW: Eu^3+^ samples. The line thicknesses represent process intensity. Dashed and wavy lines represent nonradiative transitions, while solid straight lines represent radiative ones. Please note that the energy transfer to Eu^3+^ only comes from regular WO_6_ groups—see text for explanation.

The emission (Fig. S4) and excitation spectra (see Fig. S5) were used to construct a diagram that explains the spectroscopic properties of the BMW samples obtained with the MC and CP methods (see Fig. 4). The two lowest excited energy levels of WO_6_ groups are the ^1^T_1u_ and ^3^T_1u_ multiplets^28^ and the emission results from the transition from the former to the ground state ^1^A_1g_. The position of the ^3^T_1u_ for regular and irregular WO_6_ can be determined from the components of the emission spectra at 300 K and 77 K, while the position of ^1^T_1u_ can be determined from the excitation spectra, please note that the ^1^A_1g_ → ^3^T_1u_ transition is spin forbidden so it is not perceived in the excitation spectrum. The position of the multiplets, as usual in the case of d-electron centres, depends on the position of the barycenter and the splitting of the crystal field: the larger the splitting, the lower the ^3^T_1g_ level and the higher the ^1^T_1u_. The energy distance between them is greater in the MC sample, which is probably due to a stronger crystal field than in that of the CP. The ^1^T_1u_ levels of both WO_6_ groups are well separated in the MC sample (see Fig. 4). This is why at low temperature, there is no energy transfer from regular to irregular WO_6_ for the MC sample but there is one for the CP sample. One can also expect that the energy transfer between the regular WO_6_ and Eu^3+^ will be the most efficient for the MC sample. On the other hand, the energetic proximity of the ^1^T_1u_ states of the regular and irregular WO_6_ groups in the CP sample creates a highly likely energy transfer channel that drains the regular group. As a result, less energy remains for the Eu^3+^ ions, thus their emission is weaker than for the MC samples.

The energy transfer between regular and irregular WO_6_ groups was reinforced with temperature as was observed by Blasse and Corsmit^23^. The ratio between the intensity of their emission bands describes the effectiveness of the energy transfer process from the high-energy WO_6_ group to the lower one. For the CP sample, this ratio weakly depends on temperature changing from 0.58 to 0.48 corresponding to 77 K and 300 K, respectively (Table S1). Only one emission band—from the irregular group—was observed for MC indicating that the energy transfer occurred completely in this sample. The above result indicates that the grain size and its morphology play an important role in the energy transfer process and that the transfer efficiency in the BMW: Eu^3+^ is enhanced with the increasing size.

With the increasing Eu^3+^ concentration, the intensity of the WO_6_ emission decreases, showing clearly that the excitation energy is transferred from the host to the dopant. The energy transfer to Eu^3+^ ions occurs practically only from the regular WO_6_ groups. The energy transfer mechanism is governed by the angle between W^6+^—O^2-^—Eu^3+^ bonds and is much more effective when it is equal to 180°, which is the case for the regular WO_6_. Since this angle is equal to 135° for the irregular groups, it results in a much higher transfer rate for regular WO_6_ groups compared with the irregular ones. This mechanism is discussed in more detail in the previous reports on BMW: Eu^3+^ prepared by the mechano-chemical method^10^.

To evaluate the influence of the preparation method on the efficiency of the energy transfer process from the WO_6_ groups to Eu^3+^ ions, the energy transfer efficiency $\left({\upeta }_{\mathrm{ET}}\right)$ was calculated using Eq. (1).

1 $${\eta }_{ET}=1-\frac{{I}_{S}}{{I}_{So}}$$

where I_S_, I_So_ is the integrated intensity of the sample doped with Eu^3+^ and without Eu^3+^ ions. The energy transfer between WO_6_ groups and Eu^3+^ ions was the most efficient (100% at 300 K) for the MC sample, while the lowest ${\upeta }_{\mathrm{ET}}$ value was found for the CP one (93%) (Table S2). Please note that the trend mentioned above follows the average BMW grain size which is different for distinct synthesis methods. Moreover, the higher the Eu^3+^ concentration, the shorter the distance between Eu^3+^ ions which trigger the concentration quenching of the Eu^3+^ emission. Such critical concentration *X*_*C*_ of Eu^3+^ ions depends on the synthesis methods and was 3, and 5% for MC^10^, and CP^1^, respectively.

The critical distance (R_C_) between Eu^3+^ ions can be calculated by the equation proposed by Blasse^29^:

2 $${R}_{C}=2{\left(\frac{3V}{4\pi {X}_{C}N}\right)}^{1/3}$$

where *V* is the unit cell volume, *N* is the number of chemical formula in the unit cell. In the investigated sample, *V* = 534.34 Å^3^ for CP (BMW: 5% Eu^3+^), and *V* = 534.07 Å^3^ for MC (BMW: 3% Eu^3+^). The calculated *R*_*C*_ values are presented in Table S2. Due to the fact that the value of critical distance *R*_*C*_ is larger than 5 Å, the main quenching mechanism is related to the electric multipole interaction.

**Figure 5:**
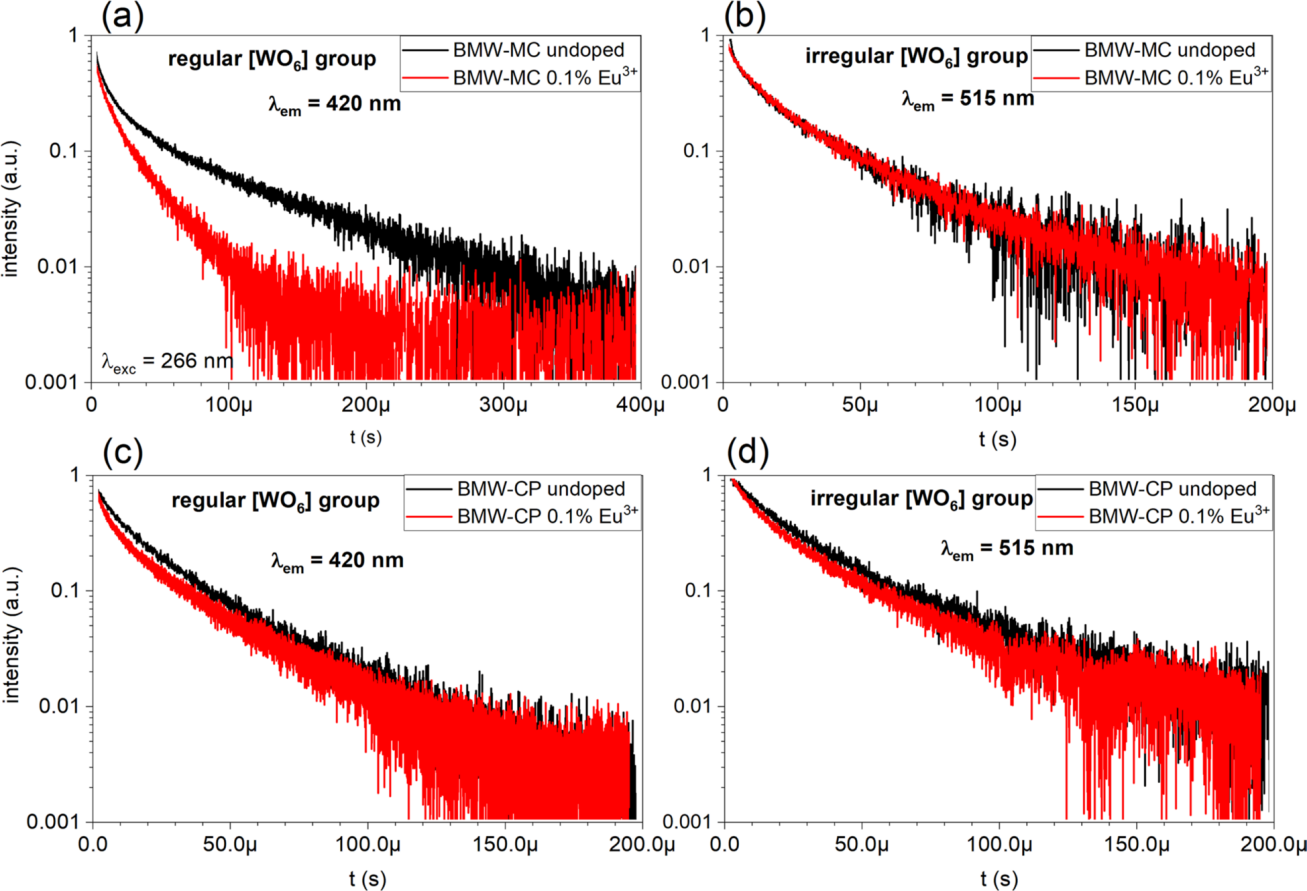
77 K luminescence decay curves of tungstate groups in doped and undoped BMW samples: MC—regular WO_6_ (**a**), MC—irregular WO_6_ (**b**), CP—regular WO_6_ (**c**), CP—irregular WO_6_ (**d**) The emission was monitored at 420 and 515 nm, for the regular and irregular WO_6_ group, respectively.

The MC sample doped with 0.1% Eu^3+^ shows a faster decay of emission from the regular WO_6_ groups (see Fig. 5a), compared to emission decay from the pure host, while the decay curve of the emission from the irregular WO_6_ groups does not depend on whether the BMW host was doped or not (see Fig. 5b). This result is an excellent demonstration that the energy transfer to Eu^3+^ occurs only from the regular WO_6_ groups. In the case of the Eu^3+^ doped CP sample, the emission decay curves of the regular and irregular WO_6_ groups differ insignificantly and the influence of the Eu^3+^ doping is much weaker, due to the much lower rate of the energy transfer (see Fig. 5c,d). As discussed earlier, the energy transfer to Eu^3+^ can occur only from the regular WO_6_ groups. This result is also consistent with the model presented in Fig. 4.

**Figure 6:**
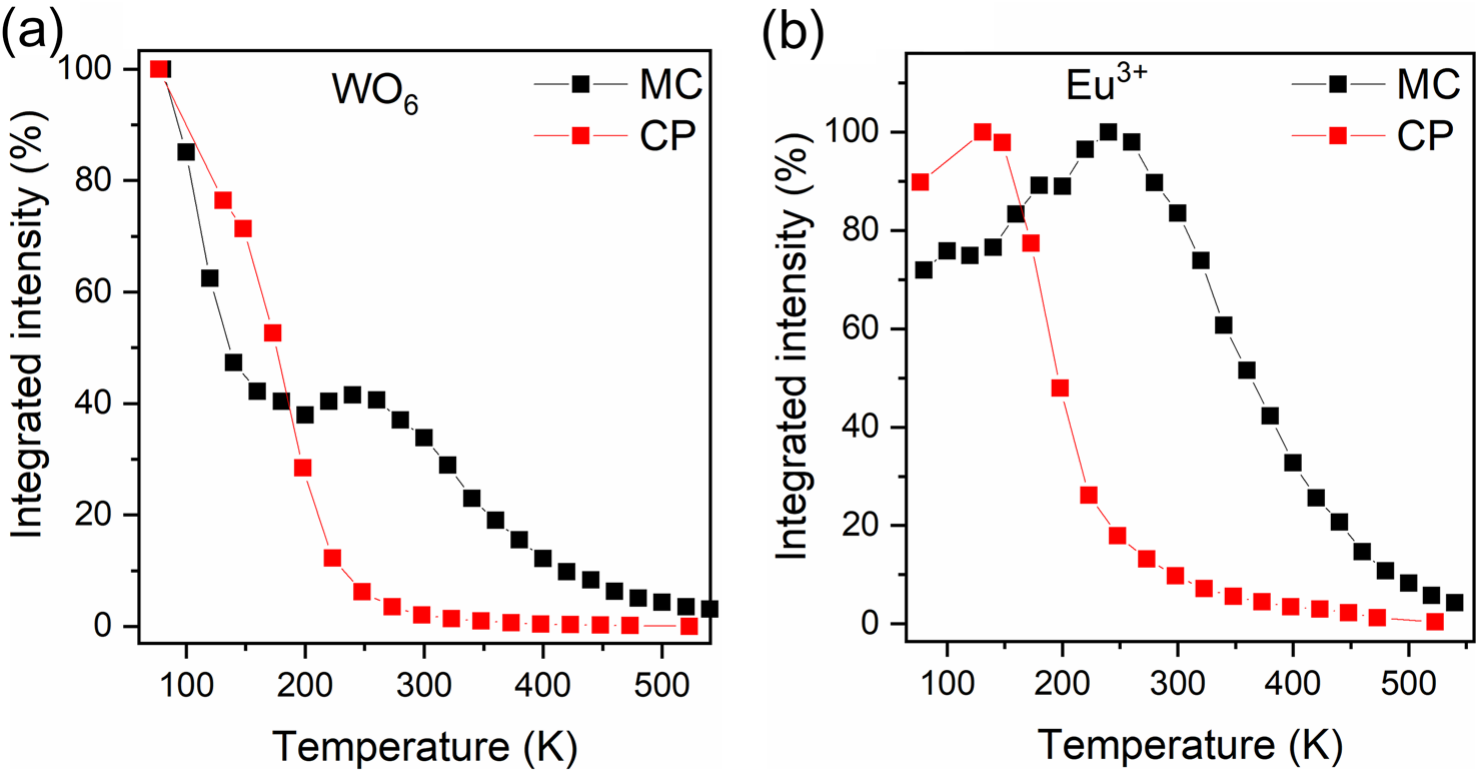
Integrated emission intensity of WO_6_ groups (**a**) and Eu^3+^ ions (**b**) of BMW: 0.1% Eu^3+^ samples prepared by MC and CP methods.

To investigate the BMW: 0.1% Eu^3+^ double perovskite as a noncontact luminescent thermometer, the temperature-dependent emission of both samples was investigated in a wide temperature range of 77–548 K (Fig S6). The temperature-dependent emission spectra of BMW: 0.1% Eu^3+^ prepared by MC have been published recently^10^. The integrated emission intensities of WO_6_ groups and Eu^3+^ ions were examined in Fig. 6. As can be seen, the emission intensity of WO_6_ groups strongly depends on synthesis routes. For better clarity, the results of the CP and MC samples were compared.

The integrated intensity of the tungstate groups of the CP sample decreases rapidly with increasing temperature and similar behaviour is observed for the MC samples up to 200 K. Above this temperature, emission intensity slightly increases due to nonradiative energy transfer between regular and irregular WO_6_ groups. After reaching the local maximum at 240 K for the MC sample, slow quenching of WO_6_ emission occurred (Fig. 6a).

The intensity of Eu^3+^ ions emission in BMW strongly depends on the synthesis condition; for the sample prepared by co-precipitation method, the emission is firmly sensitive to temperature changes beyond 150 K. For the MC sample, the emission intensity of the dopant increases with temperature due to efficient energy transfer from regular WO_6_ groups to Eu^3+^ ions. Regular quenching of emission started above the temperature of 240 K. It can be clearly seen that the stability of both WO_6_, as well as, Eu^3+^ ions strongly depends on the nonradiative interaction between them, which significantly changed with the applied synthesis method.

The thermometric parameter ∆ was defined in the following way:

3 $$\Delta \left(T\right)=\frac{{I}_{1}}{{I}_{2}}$$

where, I_1_ denotes the integrated intensity of regular and irregular WO_6_ groups emission, while I_2_ is the emission of Eu^3+^ ions. As expected, a similar profile upon temperature increase is shown by MC, the biggest changes are visible up to 170 K (Fig. S7). Whereas the profile of the CP sample has a more dynamic character in the whole temperature operating range. Due to low emission intensity, the fluctuations of ∆ parameters above 400 K are too large.

To quantify the changes of ∆ in response to temperature, the relative thermal sensitivity (S_r_) was calculated according to:

4 $${S}_{r}= \frac{1}{\Delta }\left|\frac{\partial \Delta }{\partial T}\right|$$

**Figure 7:**
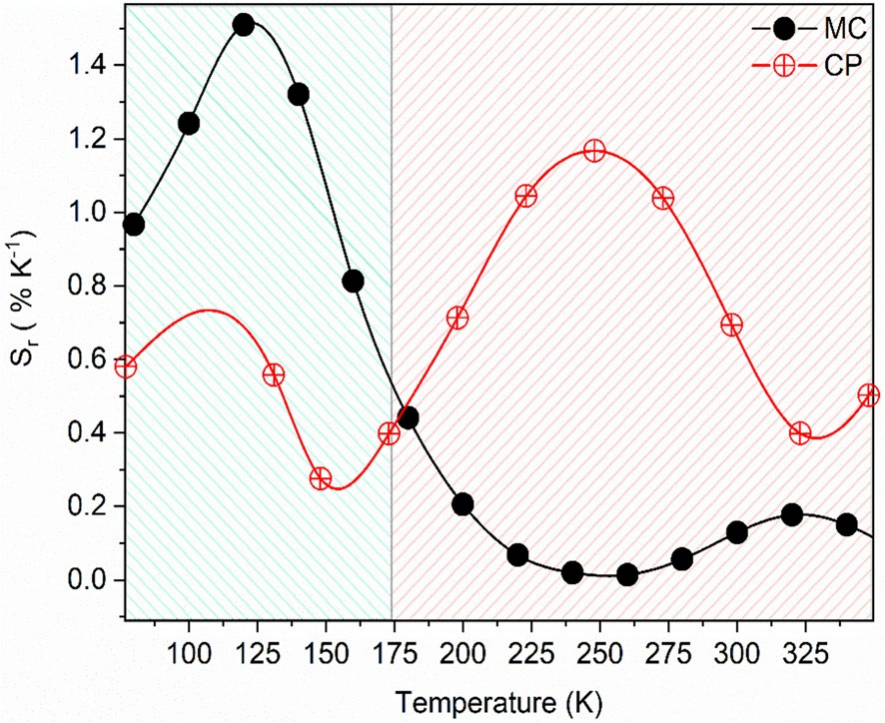
Relative thermal sensitivity values of investigated samples.

The temperature dependence of S_r_ composed of two clearly visible bands, presented in Fig. 7, resulted in two different nonradiative emission quenching channels. The first one from 80 to 175 K is related to the depopulation of WO_6_ groups emission. The maximum relative sensitivity reaches 1.5% K^−1^ at 120 K for the MC sample, whereas for CP it is closer to 0.5% K^−1^ (Table 1). However, for the CP sample the temperature at which S_r_ reaches its maximum moves to a higher temperature. The correlation between the synthesis condition and relative sensitivity of obtained noncontact luminescent thermometers seems to be evident. It can be seen that S_r_ strongly depends on energy transfer efficiency (${\upeta }_{\mathrm{ET}})$ from the regular WO_6_ group to Eu^3+^. The sensitivity of luminescent thermometers decreases with the reduction of ${\upeta }_{\mathrm{ET}}$ in the following order MC, CP. For the second region (175–350 K), the opposite trend is presented, the best sensitivity is exhibited in the CP sample with the S_r_ value of 1.17% K^−1^ at 248 K. Because of the better thermal stability of Eu^3+^ ions emission for the MC sample, the sensitivity in the 175–350 K operating temperature takes insignificant values (Fig. 7 and Table 1).

**Table 1:** Maximum value of relative sensitivity at given temperature in two: low (80–175 K) and high (175–300 K) temperature ranges.

Sample	Range 80–175 K		Range 175–300 K	
	S_r_ (%K^−1^)	Temperature (K)	S_r_ (%K^−1^)	Temperature (K)
MC	1.5	120	0.18	320
CP	0.56	130	1.17	248

In addition, it is worth comparing S_r_ with other similar materials, none of them have the maximum sensitivity at low temperature, for example, Gd_2_Ti_2_O_7_ pyrochlore with 0.46% K^−1^ (420 K)^30^; 8.52% K^−1^ (323 K) for Ca_2_MgWO_6_ co-doped Bi^3+^, Eu^3+^ phosphors^31^; 2.08% K^−1^ (398 K) and 1.51% K^−1^ (455 K) for Ba_2_LaNbO_6_ and Ca_2_LaNbO_6_ co-doped with Mn^4+^ and Eu^3+^, respectively^32^. By comparison, a luminescent thermometer based on BMW: Eu^3+^ has greater or comparable relative sensitivity. Due to such high accuracy, exceptional sensitivity as well as good physical and chemical stability, BMW: Eu^3+^ double perovskites are highly recommended for use as novel temperature sensing materials in semiconductor, medical devices, household appliances, or food processing^33^.

## Conclusions

To clarify the influence of synthesis conditions on optical properties, two synthesis methods including the mechanochemical assisted solid-state and the co-precipitation were successfully employed to synthesize Eu^3+^—activated BMW double perovskites. The different preparation conditions significantly influenced crystallinity as well as the size distribution resulting in particular types of luminescent behaviour for each sample. The MC sample has a large size distribution of the crystallites, from 1 to 6 μm, while the CP method allows to obtain small crystallites with a mean size of 200 nm and much smaller size distribution from 20 to 400 nm. In the MC samples the crystallites are agglomerated in larger particles without voids between them so they resemble pieces of dense ceramic.

The hypothesis of the presence of two W^6+^ sites in the BMW matrix was confirmed by DFT calculations. The other site is formed when W^6+^ ions take the place of Mg^2+^. This results in additional levels located below the lower edge of the conduction band. It has been shown that the relative position of the bands of the two WO_6_ groups depends on the morphology and the size of the crystallites. At 77 K, the two groups of WO_6_ are energetically isolated in the MC samples, while in CP they are not, which results in the emission of an irregular group in this latter matrix (absent in the sample MC).

A very important finding is that the excitation energy is efficiently transferred to dopant ions only from the regular WO_6_ groups. The irregular groups provide an additional channel which drains the excitation energy, resulting in the weaker emission of the dopant ions. This is particularly visible for the CP matrix for which the transfer from the regular group to the irregular one is very intense. This is also confirmed by the analysis of emission decay profiles for the Eu^3+^ and WO_6_ groups, indicating no transfer from irregular groups and almost 100% transfer from the regular ones to the dopant ion for the MC sample. The general conclusion is that to ensure the intensive dopant emission of BMW: Eu^3+^, the host must be synthesized by the MC method and the Li^+^ co-dopant provides local charge compensation and prevents concentration quenching.

However, in two other respects, the CP samples are superior to the MC samples. Firstly, CP has a greater homogeneity of crystallite sizes, which together with the nanometric size, makes it a candidate for obtaining transparent ceramics. Secondly, CP samples have proven to have enormous potential for luminescent thermometric applications. The relative sensitivity of temperature readout was successfully manipulated by the synthesis routes. In the temperature range of 150–325 K, the CP sample was more sensitive to temperature fluctuation than others. However, for low-temperature detection of 80–175 K, the highest capabilities for temperature readout still belongs to the MC sample with S_r_ of 1.5% K^−1^ at 120 K.

## Experimental method

### Synthesis

In this study, representative samples of BMW: x % Eu^3+^ (x = 0, 0.1%, 5%) were successfully obtained by two different methods: co-precipitation^1^, and mechanochemical assisted by the high-energy milling method^10^. All samples were annealed. All necessary information about the synthesis, such as chemical precursors, grinding and annealing time, and temperature, has been published recently^1,10^.

### Measurements and characterization

The X-ray powder diffraction patterns of some representative samples of BMW: Eu^3+^ were obtained using an X’Pert ProPANalytical X-ray diffractometer by means of Cu Kα radiation (λ = 1.54056 Å) in a 2 $\theta$ range from 10º to 90º with a step size of Δ2θ = 0.02. The 77 K, 300 K emission spectra of all samples were measured applying a Hamamatsu Photonic multichannel analyser PMA^−1^2 along with a BT-CCD linear image sensor. All emission spectra were corrected for the spectral characteristics of the monochromator and the sensitivity of the detector as a function of wavelength. The emission decay profiles were recorded with a Lecroy digital oscilloscope using an excitation source of Nd: YAG. The thermal quenching measurements were performed using a Hamamatsu Photonic multichannel analyser PMA-12 along with a BT-CCD linear image sensor equipped with the Linkam THMS 600 Heating/Freezing Stage to control the temperature of the samples.

### Computational details

Electronic structure calculations were performed with the use of the VASP package^34,35^. The Perdew–Wang parameterization of the local density approximation (LDA^36^) was employed. The spin–orbit coupling was included. A plane-wave energy basis with a cut-off energy of 500 eV and a 7 × 7 × 7 k-point grid were applied. The systems were modelled with cubic supercells of 80 ions (the 2 × 2 × 2 multiplication of a primitive unit cell), in which a Mg-W antisite defect was a local substitution of Mg and W ions. The lattice parameters and all atomic positions were relaxed.

## Supplementary Information

Supplementary Information.
