# Supplementary material for: On how the mechanochemical and co-precipitation synthesis method changes the sensitivity and operating range of the Ba2Mg1-xEuxWO6 optical thermometer
Source: Sci Rep. 2021 Nov 24;11:22847. doi: 10.1038/s41598-021-02309-9 (PMC8613287; doi:10.1038/s41598-021-02309-9)
Supplement: Supplementary file 1 — Supplementary Information. [file 41598_2021_2309_MOESM1_ESM.docx]

**Supplementary information**

**On how the mechanochemical and co-precipitation synthesis method changes the sensitivity and operating range of the**

**Ba_2_Mg_1-x_ Eu_x_WO_6_ optical thermometer**

T. H. Q. Vu, Bartosz Bondzior, Dagmara Stefańska*, Natalia Miniajluk-Gaweł, Maciej J. Winiarski, Przemysław J. Dereń*

Institute of Low Temperature and Structure Research, Polish Academy of Sciences, Okólna 2, 50-422 Wrocław, Poland

* Corresponding authors: Przemysław J. Dereń ([p.deren@intibs.pl](mailto:p.deren@intibs.pl)) and Dagmara Stefańska (d.stefanska@intibs.pl)





**Figure S1.** a) X-ray powder diffraction lines of BMW : 5% Eu^3+^ synthesized by co-precipitation (red line), mechanochemical method (blue line) and b) Enlargement of the highest diffraction peak.





**Figure S2.** Crystallite size distribution of some representative samples of BMW : 5 % Eu^3+^ synthesized by co-precipitation (red), mechanochemical method (blue).





**Figure S3.** SEM images of BMW : 5 % Eu^3+^ synthesized by CP (a, b, c, d, e, f) and MC method (g, h, i, j, k, l) with different magnifications





**Figure S4.** Emission spectra of BMW : 5 % Eu^3+^ synthesized by CP (red), MC (blue) recorded under 266 nm excitation at 300 K (a) and 77 K (b).





**Figure S5.** Excitation spectra of BMW : 5 % Eu^3+^ synthesized by CP (a), MC (b) monitored at 425 nm at 77 K (grey line) and 525 nm at 300 K (black line). The dashed lines represent the Gaussian fitting peaks.

**Table S1.** The ratio of emission intensity between regular and irregular [WO_6_]^6-^ groups of undoped BMW synthesized by the co-precipitation and mechanochemical method at 77 K and 300 K.

| **Temperature (K)** | **CP** | **MC** |
| --- | --- | --- |
| 77 K | 0.58 | 0.9 |
| 300 K | 0.48 | 0.17 |

**Table S2.** The energy transfer efficiency 𝜂 (left: at 77 K, right: at 300 K), the concentration quenching X_c_ and the critical distance R_c_ of BMW : x% Eu^3+^ (x = 0; 5 %) synthesized by the co-precipitation and mechanochemical method.

| **Sample** | **𝜂 (%)** | **X_C_** | **R_C_** |
| --- | --- | --- | --- |
| **CP** | 91/93 | 5 | 17.22 |
| **MC** | 100/100 | 3 | 20.41 |





**Figure S6.** Temperature – dependent emission spectra of BMW: 0.1 % Eu^3+^ prepared by the co-precipitation (a) and mechanochemical method (b).^[[1]](#footnote-1)^





**Figure S7.** Changes of thermometric parameter ∆ with temperature for MC (black) and CP (blue) samples. The dashed line marks the beginning of zone out of operating temperature range.

1. The figure S6 b was published in Journal of Alloys and Compounds, 842, D. Stefańska, B. Bondzior, T.H.Q. Vu, N. Miniajluk-Gaweł, P.J. Dereń, The influence of morphology and Eu^3+^ concentration on luminescence and temperature sensing behavior of Ba_2_MgWO_6_ double perovskite as a potential optical thermometer, 155742, Copyright Elsevier (2020).” [↑](#footnote-ref-1)
